# Membranoproliferative Glomerulonephritis and X-Linked Agammaglobulinemia: An Uncommon Association

**DOI:** 10.1155/2014/480947

**Published:** 2014-03-04

**Authors:** Vasco Lavrador, Filipa Correia, Rita Sampaio, Cristina Cândido, Maria Sameiro-Faria, Laura Marques, Conceição Mota

**Affiliations:** ^1^Pediatric Infectious Diseases and Immunodeficiencies Unit, Centro Hospitalar do Porto, Largo Professor Abel Salazar, 4099-001 Porto, Portugal; ^2^Department of Pathology, Centro Hospitalar do Porto, Largo Professor Abel Salazar, 4099-001 Porto, Portugal; ^3^Department of Pediatrics, Centro Hospitalar de Trás-os-Montes e Alto Douro, Avenida Noruega, 5000-508 Vila Real, Portugal; ^4^Pediatric Nephrology Department, Centro Hospitalar do Porto, Largo Professor Abel Salazar, 4099-001 Porto, Portugal

## Abstract

*Introduction*. X-linked agammaglobulinemia (XLA) is a primary immunodeficiency characterized by agammaglobulinemia requiring replacement treatment with immunoglobulin. The association of XLA and membranoproliferative glomerulonephritis (MPGN) is unexpected and, to our knowledge, only one case was previously published. *Case Report*. The authors report the case of a 10-year-old boy with family history and prenatal diagnosis of XLA, treated from birth with intravenous immunoglobulin replacement therapy. He presented with pneumonia, macroscopic hematuria, nephrotic proteinuria, hypoalbuminemia, and hypercholesterolemia with normal renal function and serum complement levels. Renal histology showed immune complex mediated MPGN. He was started on high dose prednisolone and ramipril and switched to weekly subcutaneous immunoglobulin. After a 4-month treatment, hematuria and proteinuria significantly improved and prednisolone was gradually tapered without relapse. *Conclusion*. The pathogenic process underlying MPGN development in this patient is unknown but residual humoral immunity might play an important role. Thus, this case highlights the risk of autoimmune disorders among patients with XLA.

## 1. Introduction

Membranoproliferative glomerulonephritis (MPGN) is an uncommon cause of chronic nephritis that occurs primarily in children and young adults. In most cases, complement activation is prominent and immune complex formation and deposition in the glomeruli play an important role [1–3]. On the other hand, patients with X-linked agammaglobulinemia (XLA, MIM number 300755), also known as Bruton agammaglobulinemia, have a profound defect in B-lymphocyte development resulting in severe hypogammaglobulinemia. Bruton tyrosine kinase (BTK) gene mutation on the X chromosome has been identified as responsible for this disease. Replacement therapy with regular administration of intravenous (IVIG) or subcutaneous (SCIG) immunoglobulin is the only effective treatment, preventing severe infections, which are the hallmark of the untreated disease [4].

The authors report the case of a patient with XLA who developed MPGN during treatment with IVIG. The association of XLA and MPGN is unusual and, to our knowledge, only one case was previously reported [5], although other cases with renal immune complex deposition were also described [6] and are listed in Table 1.

## 2. Case Presentation

The patient in this report has a family history of XLA with two affected siblings, with their mother being a carrier of the mutation. He was screened prenatally and the genetic analysis revealed a BTK mutation (R288Q). A masculine gender newborn was born after a full-term pregnancy with a normal delivery, weighing 3250 grams, with an otherwise unremarkable neonatal history. He was started on IVIG shortly after birth. He showed low but detectable levels of serum IgG (1.85 g/L; reference range 7.24–1591), IgA (59 mg/L; reference range 900–3320), and IgM (70 mg/L; reference range 520–2080) with CD19+ 1.4% and CD 20+ 1.6% at the age of 8 months. Although the patient had a poor compliance with medical follow-up, including IVIG treatment, there was no record of major infections during childhood. At the age of 10 years, he was admitted with right lower lobe pneumonia and macroscopic hematuria. Physical examination revealed normal blood pressure and no peripheral edema. Initial laboratory evaluation showed hypoalbuminemia (27 g/L; reference range 32–45), hypercholesterolemia (5.72 mmol/L; high >5.18) and normal renal function with serum creatinine of 32.71 *μ*mol/L (reference range 46.9–69.8) and estimated glomerular function rate of 2.62 mL/s/m^2^. Urinalysis revealed proteinuria (urinary protein over creatinine ratio of 632.8 mg/mmol) with the following urinary sediment: erythrocytes 50 per high power field (HPF), leucocytes 5–10/HPF, and red blood cells and hialines casts 1-2/HPF. Normal serum complement levels were observed, with C3 level of 1.51 g/L (reference range 0.81–1.67) and C4 of 0.32 g/L (reference range 0.11–0.41). The etiological diagnosis of pneumonia could not be established.

**Figure 1:**
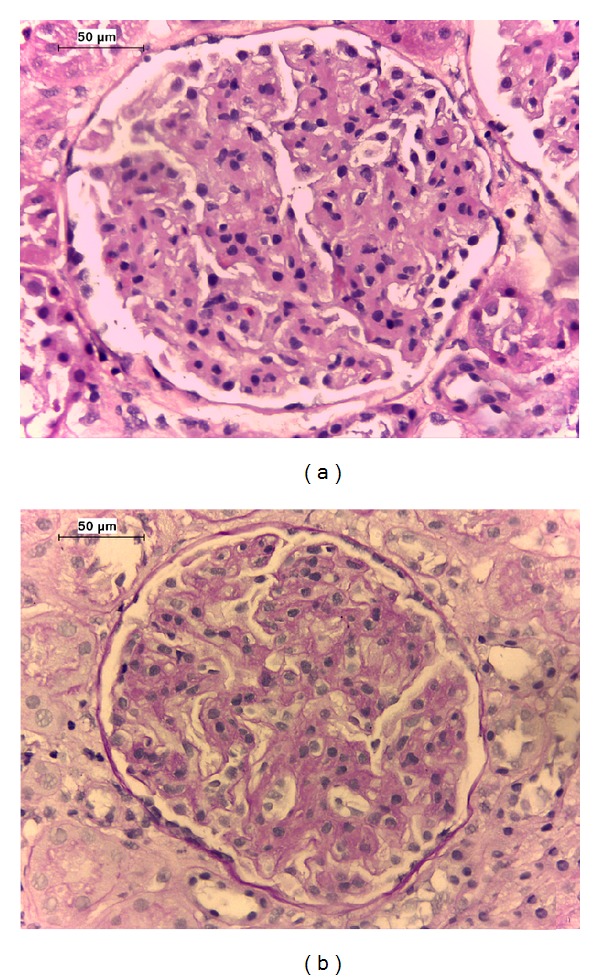
Histology of the patient renal biopsy. (a) Proliferation of mesangial and endothelial cells and expansion of the mesangial matrix (hematoxylin and eosin stained section; original magnification ×400); (b) global endocapillary proliferation and a double contour appearance of the glomerular basement membrane (periodic acid-Schiff stained section; original magnification ×400).

Further investigation disclosed normal autoantibodies serum levels and excluded HIVs 1 and 2, HBV, and HCV infection, with negative viral load and normal liver enzymes. A renal biopsy was performed and histology presented glomeruli with membranoproliferative patterns, without significant interstitial inflammation or tubular atrophy (Figure 1). Immunofluorescence study revealed glomerular deposition of IgG (+++), C3 (++), C4 (+), C1q (++), and traces of IgM in the mesangium and subendothelial space. The patient was diagnosed with MPGN type I, reclassified as immune complex mediated glomerulonephritis according to a recent classification [3].

IVIG was switched to SCIG replacement therapy in a weekly regimen and prednisolone was also started at an initial dose of 2 mg/kg/day, which was gradually tapered. Ramipril was also started at a dose of 2,5 mg/day. After 18 months the patient maintained a normal blood pressure and normal renal function. The urinary sediment is near normal (erythrocytes 5–10/HPF, leucocytes 0–2/HPF) and no proteinuria was detected (urinary protein/creatinine ratio of 20,34 mg/mmol). There were no major infections and he maintained an adequate growth (weight and stature z-scores of 0.81 and 0.31 at age of 12 years).

## 3. Discussion

The classic XLA phenotype is characterized by a reduced peripheral B-lymphocyte count and near complete absence of endogenous immunoglobulin. More than 500 different BTK mutations have been reported in patients with XLA but a consistent correlation between genotype and phenotype remains to be established [7]. Given this low level of immunoglobulin, XLA patients should not be prone to immune complex mediated disorders. Even so, this patient had a MPGN characterized by immune deposits in the mesangium and subendothelial space, which is thought to reflect the deposition of circulating immune complexes [1].

Immune complex-mediated MPGN is most commonly secondary to viral infections (hepatites C and B) and less frequently to a chronic bacterial disease (e.g., endocarditis, shunt nephritis, and abscesses). Although the patient had a poor compliance with immunoglobulin treatment, no important infections were noted until the age of 10 years. Autoimmune diseases like systemic lupus erythematosus (particularly in the chronic phase of lupus nephritis) and, less often, Sjögren's syndrome or rheumatoid arthritis are also associated with MPGN. The patient had neither clinical manifestations nor autoantibodies compatible with these multisystemic disorders. Monoclonal gammopathy is another cause of secondary MPGN but is an extremely rare diagnosis in childhood and no laboratory findings support it. So, like reported in another XLA patient with MPGN, the most frequent secondary causes of MPGN were excluded, but a viral etiology is still to be suspected [1, 5]. The preparation of immunoglobulin was also replaced by a different brand and switched to subcutaneous administration, making it unlikely for immune complexes to be formed through the reaction of IVIG with an endogenous antigen; the most likely hypothesis is that they were formed by the reaction between the patients endogenous residual IgG and an endogenous antigen. Although we cannot ascertain the precise level of innate immunoglobulin because IVIG was started shortly after birth, this patient exhibits detectable levels of IgA and IgM and a greater percentage of B-cells than most patients with XLA, which might imply a higher production of endogenous IgG. Besides, the improvement following corticosteroid therapy also supports that residual humoral immunity might be causally related to the development of MPGN. It remains unclear whether an association between XLA and MPGN (or other forms of renal immune disease) can be established and the underlying pathophysiologic mechanisms are still not fully understood. However, there is growing evidence that patients with primary immunodeficiencies may also be prone to develop autoimmune disorders due to an imbalance in immunoregulatory pathways [8].

## Figures and Tables

**Table 1:** Patients with XLA and renal disease due to immune complex deposition.

Reference	Age	Presentation	Renal histology	Treatment	Outcome
Present case	10 years	Pneumonia, macroscopic hematuria, and nephrotic proteinuria	MPGN type I	Switched immunoglobulin brand; corticosteroid therapy	Asymptomatic with normal renal function and no proteinuria and improved hematuria

Yoshino et al., 2006 [5]	3 years	Microscopic hematuria, nephrotic proteinuria, and low serum complement	MPGN type III	Switched immunoglobulin brand; corticosteroid therapy	Asymptomatic with normal renal function and improved hematuria and proteinuria

Endo et al., 2011 [6]	23 years	Microscopic hematuria	Membranous glomerulopathy	Switched immunoglobulin brand	Asymptomatic with normal renal function and minimal proteinuria
